# Supervised Injecting Room Cohort Study (SIRX): study protocol

**DOI:** 10.1136/bmjopen-2024-091337

**Published:** 2025-02-11

**Authors:** Ashleigh C Stewart, Matthew Hickman, Paul A Agius, Nick Scott, Jack Stone, Amanda Roxburgh, Daniel O’Keefe, Peter Higgs, Thomas Kerr, Mark A Stoové, Alexander Thompson, Sione Crawford, Josephine Norman, Dylan Vella-Horne, Zachary Lloyd, Nico Clark, Lisa Maher, Paul Dietze

**Affiliations:** 1 Disease Elimination, Burnet Institute, Melburne, Victoria, Australia; 2 School of Public Health and Preventive Medicine, Monash University, Melbourne, Victoria, Australia; 3 Population Health Sciences, University of Bristol, Bristol, UK; 4 National Drug and Alcohol Research Centre, Sydney, New South Wales, Australia; 5 Deakin University Faculty of Health, Burwood, Victoria, Australia; 6 Public Health, La Trobe University, Bundoora, Victoria, Australia; 7 Division of Social Medicine, The University of British Columbia Department of Medicine, Vancouver, British Columbia, Canada; 8 British Columbia Centre on Substance Use, Vancouver, British Columbia, Canada; 9 Department of Gastroenterology, St Vincent’s Hospital Melbourne, Fitzroy, Victoria, Australia; 10 Department of Medicine, The University of Melbourne, Melbourne, Victoria, Australia; 11 Harm Reduction Victoria, Melbourne, Victoria, Australia; 12 Centre for Evaluation and Research Evidence, Department of Health Victoria, Melbourne, Victoria, Australia; 13 The Kirby Institute, Kensington, New South Wales, Australia; 14 National Drug Research Institute, Melbourne, Victoria, Australia

**Keywords:** Drug Utilization, Health Services, Substance misuse

## Abstract

**Abstract:**

**Background:**

Supervised injecting facilities (SIFs) are designed to reduce the harms associated with injecting drug use and improve access to health and support services for people who need them. The Supervised Injecting Room Cohort Study (SIRX) aims to provide evidence of the effects, including cost-effectiveness, of a SIF embedded within a community health service, the Melbourne Medically Supervised Injecting Room (MSIR), which has a range of integrated harm reduction, health and social support services on-site.

**Methods and analysis:**

The SIRX study design involves two prospective cohort studies that collect behavioural data and retrospectively and prospectively linked administrative data for primary and tertiary health services, criminal justice records, and mortality. The two cohorts are: (1) participants drawn from the existing Melbourne Injecting Drug User Cohort Study (SuperMIX; established in 2008–ongoing) through which participants consent to annual behavioural surveys (including serological testing for HIV and hepatitis B and C viruses) and linkage to administrative data; and (2) the SIRX-Registration Cohort (SIRX-R; established in 2024) comprising registered MSIR clients who consent to a baseline behavioural survey and administrative data linkage including the frequency of SIF use, and the uptake of on-site services. Primary outcomes are aligned to the legislated aims of the Melbourne MSIR, including ambulance-attended non-fatal overdoses and all-cause and drug-related mortality. Using causal inference methods, analyses will estimate the effect of MSIR exposure (frequent use/infrequent use/no use) on these primary outcomes. The SIRX study also has a secondary focus on the effect of MSIR exposure on health service use and related outcomes.

**Ethics and dissemination:**

SuperMIX Study (599/21) and SIRX-R Study (71/23) ethics approvals were obtained from Alfred Hospital Research Ethics Committee. Participants will be assessed for capacity to provide informed consent following a detailed explanation of the study. Participants are informed of their right to withdraw from the study at any time and that withdrawing does not impact their access to services. Aggregated research results will be disseminated via presentations at national and international scientific conferences and publications in peer-reviewed journals. Local-level reports and outputs will be distributed to key study stakeholders and policymakers. Summary findings via accessible outputs (eg, short infographic summaries) for participants will be displayed in relevant services including the Melbourne MSIR and the study van, and distributed via Harm Reduction Victoria.

**STRENGTHS AND LIMITATIONS OF THIS STUDY**

- The Supervised Injecting Room Cohort Study (SIRX) uses a cohort and quasi-experimental design to measure varying levels of exposure to the Melbourne Medically Supervised Injecting Room (MSIR) and its on-site services and the effect of these exposure levels across a range of outcomes.
- Comprehensive longitudinal behavioural data and linkage to MSIR visits and routinely collected administrative health and social databases.
- The SuperMIX Cohort may be subject to attrition bias through lost-to-follow-up; however, data linkage for primary outcomes minuses this risk.
- Consistent with all observational studies, confounding may impact the observed associations, but casual inference methods are being applied to minimise this.

## Background

 Regular injecting drug use is associated with a wide range of adverse health outcomes including drug overdose deaths, which have steadily increased in the last two decades in Australia and globally.^1 2^ In particular, opioid-related deaths in Australia have doubled since 2001.^3^ Injecting-related harms also include blood-borne viral infections (eg, HIV, viral hepatitis) and injection-related injuries and infections (eg, skin, soft tissue injuries and endocarditis),^1 4^ all of which are key drivers of morbidity, mortality, as well as drug-related economic costs to the community.^5 6^ Injecting drug use is also connected to wider social and economic harms, including drug-related crime^7^ and avoidable healthcare costs,^8 9^ many of which are related to current drug policy and the criminalisation of illicit drugs.^10^

Social marginalisation, stigma and trauma are driven by myriad factors including adverse childhood experiences,^11 12^ unemployment,^13^ persistent housing instability,^14 15^ and imprisonment^4 16 17^ and compound the risks among people who inject drugs.^18 19^ Stigma and discrimination, in particular, contribute to suboptimal healthcare, undermining access to supportive and harm reduction services, such as opioid agonist therapy (OAT).^20^ Further, harms created by structural barriers and social exclusion can cause delays in seeking care, which may lead to frequent use of acute care services such as hospital emergency departments (ED).^21^

Harm reduction interventions, including needle and syringe programmes (NSPs) and OAT, have been shown to be effective at reducing injecting-related harm^22^ and health service use^23^ but programme coverage of these interventions is variable in Australia and may be insufficient to reduce drug-related harms.^24^ Further, NSPs and OAT do not provide an immediate response to acute harms, such as drug overdoses and the costs associated with managing them.

### Supervised injecting facilities

Supervised injecting facilities (SIFs; many labels are used to describe supervised drug consumption sites and overdose prevention centres but as this article is focused on facilities that only permit drug injecting under supervision, the term ‘supervised injecting facility’ is used throughout the article) were first established in Europe in the mid-1980s in response to epidemics of public injecting, overdose, and increasing HIV incidence related to injection drug use,^25^ and as of 2023, SIFs were legally operating in 17 (predominantly high income) countries.^24^ SIFs, and drug consumption rooms more broadly, provide an environment where individuals can use pre-obtained drugs with sterile equipment under supervision. SIFs provide an emergency response in the event of drug overdose, facilitate referrals to other health and social service providers, and sometimes, also provide a range of on-site services. While referrals from SIFs have been shown to result in the uptake of services,^26^ people who use SIFs have also demonstrated a preference to receive care on-site, due to the relationship with SIF staff, and negative experiences with mainstream health services.^27^

Evidence from observational, modelling, ecological and qualitative studies demonstrates that SIFs reduce a range of harms, including drug-related^27^,^29^ and all-cause mortality,^30^ ambulance attendances for drug overdose,^31^ and ED presentations.^32 33^ Evidence also suggests SIFs are associated with reductions in experiencing violence,^34 35^ receptive needle and syringe sharing,^36^ and HIV incidence.^37^ Further, SIFs have demonstrated effectiveness in attracting individuals at greatest risk of harm, such as those experiencing homelessness, people with mental illness, people who inject in public spaces and people who engage in high-risk drug use.^38^,^40^ SIFs have had a documented positive impact on public amenity by reducing public injecting and ensuring safe disposal of injecting equipment.^41 42^ Thus, SIFs demonstrate utility in offering a range of harm reduction services and referrals to populations considered to be experiencing social and structural vulnerability.

Despite this evidence base, the evaluation of SIFs remains challenging with researchers determining randomised control trials to be difficult to implement and unethical for evaluating the effect of SIFs, particularly in the absence of clinical equipoise (uncertainty over evidential strength for an intervention) when intervening in the event of an overdose.^43 44^ Thus, past evaluations have relied exclusively on observational data, which creates methodological challenges and when considering hierarchies of evidence. Further, the vast majority of international evidence in a recent review (16/22, studies, 72%) relates to a single SIF in Vancouver, Canada (Insite),^45^ itself a unique risk environment for drug-related harm characterised by epidemics of HIV infection and overdose.^46^ Thus, there remains an ongoing need to evaluate the impacts of SIFs with many unanswered questions, as well as a need to evaluate SIFs operating in other international contexts, with different operational models, and varying drug markets, patterns of drug use and epidemiological environments. For example, previous SIF cost-effectiveness studies are focused on reductions in HIV incidence, which is of limited relevance to jurisdictions such as Australia where HIV remains rare among people who inject drugs.^47 48^

### The Melbourne medically supervised injecting room (MSIR)

The Melbourne Medically Supervised Injecting Room (MSIR) was established in 2018, in North Richmond, an inner-city suburb of Melbourne with an established street drug market.^49^ The North Richmond MSIR is located in the grounds of the largest public housing estate in Australia, directly adjacent to the North Richmond Community Health Centre. Prior to the establishment of the Melbourne MSIR, local drug market activity in the North Richmond area was characterised by highly visible public injecting, injecting-related litter and high rates of fatal and non-fatal drug overdoses.^49^,^51^ Within the first 18 months of operation, approximately 4000 clients registered to use the MSIR, with the facility averaging 300 visits per day following the opening of the purpose-built facility.^50^

Based on consultations with people injecting drugs in the local area, the Melbourne MSIR was designed to meet their needs as a ‘one-stop shop’, incorporating an extensive range of on-site health and social services,^52^ including blood-borne virus testing and treatment,^53 54^ OAT,^55^ oral healthcare, housing support, wound care, mental health support, legal assistance, food, and primary care, as well as referral to other services when needed. The design of each of the health and social services was optimised to be responsive to the needs of the people who inject drugs, by offering simplified treatment pathways, incorporating increased flexibility in service delivery and using a trauma-informed approach. This resulted in a substantial uptake in on-site services, including 387 treatment initiations for hepatitis C, and 1096 initiations of OAT,^56^ in some ways distinguishing the MSIR from other SIFs.

In 2020, an MSIR service review assessed the facility against its legislated objectives to: (i) reduce avoidable deaths caused by drug overdose; (ii) advance delivery of effective health services to MSIR clients; (iii) reduce attendance and use of emergency and hospital services for drug overdose; (iv) reduce discarded needles in public places and public injecting; (v) improve neighbourhood amenity (this was not defined but typically relates to public injecting, public overdose, discarded injecting equipment and perceived safety of the surrounding environment^57^); and (vi) reduce blood-borne virus transmission.^50^ Based on two reviews in 2020 and 2023, noted reductions in preventable deaths (including management of almost 6000 overdose events) and use of emergency services for overdose, and improvements in public amenity were observed.^50 58^ Thus, the government extended the MSIR operating license for 3 years and recommended the establishment of a second facility in the Melbourne Central Business District.^59^ In 2024 following strong resistance from business owners and residents, driven by ongoing negative media and concerns about potential increases in crime and impacts on property values,^60^,^62^ as well as the failure to establish a suitable location,^63^ the commitment to establish the second facility was withdrawn.^64^

Despite these MSIR reviews demonstrating reductions for each of the facility-specific legislated aims, data used to generate these estimates were limited by small sample sizes of MSIR clients, coupled with a short evaluation timeframe that reduced analytical power. The withdrawal of the commitment to establish a second facility highlights the precarious situation of SIFs and the need for robust evidence on the effects of SIFs, particularly in light of suggestions by the Victorian opposition that they would close the MSIR if elected.^65^ To this effect, the Supervised Injecting Room Cohort Study (SIRX) is designed as a large-sample longitudinal cohort study to provide the strongest possible evidence of the effects of the MSIR including its on-site services model. This protocol paper describes our approach to evaluating the impacts of the Melbourne-based SIF, focused on the facility’s legislated aims (outlined below) using comprehensive longitudinal data from two cohort studies of people who inject drugs.

### The Melbourne injecting drug use cohort study (SuperMIX)

Data from The Melbourne Injecting Drug User Cohort Study (SuperMIX) were used to inform part of the MSIR facility reviews. SuperMIX is the largest, only active and longest running longitudinal study of people who inject drugs in Australia and one of the largest internationally (n>1500 enrolled participants).^16^ Established in 2008, SuperMIX involves annual interviews collecting detailed data on drug use and risk behaviours, drug purchasing, health service and drug treatment utilisation, health and well-being, and imprisonment. These detailed data are complemented by serological testing for HIV and viral hepatitis, and linkage to the National Death Index (NDI), National Medical and Pharmaceutical Benefits Schemes (MBS, PBS), state-wide emergency department presentations and hospitalisations, ambulance attendances, and drug treatment contacts.^16^

Following the MSIR opening, SuperMIX participants were asked about their level and frequency of exposure to the MSIR, annually. As part of the MSIR facility review, SuperMIX findings demonstrated that MSIR clients were more likely to identify as Aboriginal and/or Torres Strait Islander, report recent arrest and report heroin as their main drug of choice.^50^ SuperMIX participants previously reporting injecting in high-risk settings, such as in public spaces, were almost twice as likely to visit the MSIR compared with participants not injecting in these settings. The MSIR review also found MSIR use was associated with lower rates of ambulance attendance and naloxone administration, but these findings were drawn from small samples or limited ecological data.^50^

The current study will leverage the existing infrastructure of SuperMIX, particularly the study’s established relationships with people who inject drugs and the MSIR service.

### The current study

The SIRX involves a breadth of longitudinal data obtained linked to administrative health and social databases. With annually measured SuperMIX prospective cohort data linked via MSIR facility registration, SIRX presents the opportunity to implement a quasi-experimental study design permitting estimation of the causal effect of MSIR exposure (including both safe injecting and the use of on-site services) using causal inference methods. This study design was successfully applied in Vancouver,^66^ providing the strongest evidence to date of SIF effectiveness.^45^ SIRX will broaden the evidence base for SIFs to inform harm reduction responses to reduce injecting-related harms particularly in Australia given opioid-related deaths have doubled since 2001.

SIRX aims to provide further evidence of the effects, including cost-effectiveness, of SIFs by estimating the total causal effect of MSIR use on all-cause and opioid-related mortality and non-fatal overdose. This study design will also allow for new evidence on additional secondary outcomes related to SIF use such as self-reported public injecting, public syringe disposal and receptive syringe sharing, and enhanced hepatitis C treatment and health protective behaviours including vaccination (eg, COVID-19 and hepatitis B). It will also allow for a cost-effectiveness analysis to be undertaken that combines a broader set of MSIR benefits, which have not previously been able to be quantified.

### Aims

The primary aim of the SIRX study is to estimate longitudinal associations and any causal effects between MSIR use and non-fatal overdose and mortality, and linked secondary aims are to examine similar effects on additional outcomes, including tertiary health service and drug treatment uptake. The study will also evaluate the cost-effectiveness of the MSIR service in relation to these aims.

## Methods

### Study setting & design

**Figure 1:**
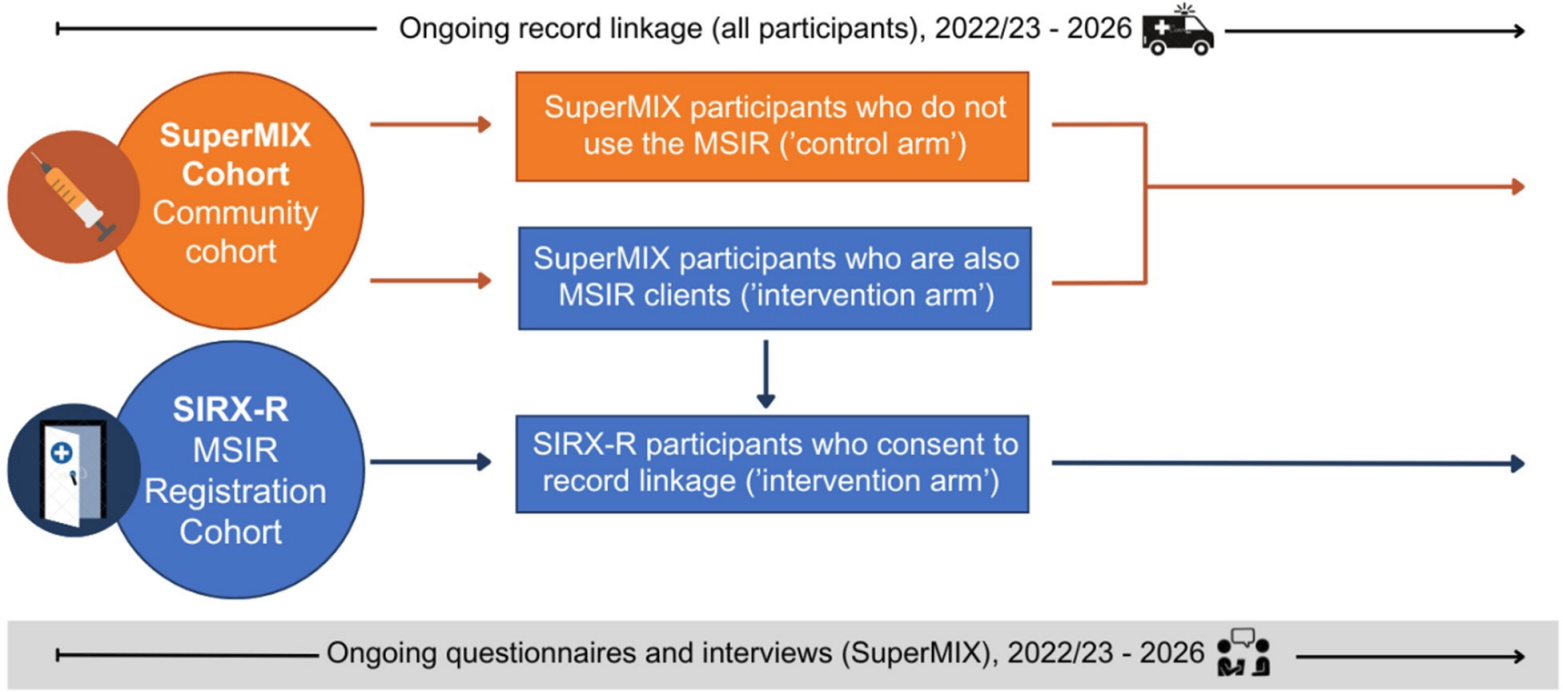
Study design of the SIRX Study.

The SIRX Study will be undertaken across Melbourne, Australia, with a particular focus on North Richmond, an inner suburb of Melbourne where the MSIR is located. The SIRX Study uses a cohort and quasi-experimental design in which MSIR clients and people who inject drugs with varying levels of MSIR exposure are compared across a range of behavioural and linked administrative outcome data. Two cohorts will contribute to the SIRX Study, the SuperMIX Cohort and the SIRX-Registration (SIRX-R) Cohort (figure 1).

### Participant eligibility

MSIR clients will be eligible for the SIRX Study if they attend and use the MSIR in the 6 months prior to study contact and consent to an interview and record linkage. Participants also provide unspecified consent for their data to be used in other ethically approved studies involving project investigators. Currently, MSIR clients must be aged 18+ years and have initiated injecting drugs prior to MSIR registration and are neither on parole nor pregnant; SIRX Study eligibility will align with these requirements, which are similar to SuperMIX eligibility.^16^ Clients provide minimal information when registering and are given an anonymous client number, allowing for internal tracking on an MSIR database. Data on the utilisation of on-site health and social services is incorporated into the analysis.

### Study recruitment

A total of 3000 registered MSIR clients will be recruited into the SIRX Study. MSIR data show approximately 3600 individual clients access the MSIR every 9 months, indicating the need to recruit every second client to complete recruitment in the expected 9-month recruitment period. Previous experience indicates rapid recruitment of a large number of participants using these methods.^66^ Recruitment inside the MSIR will be led by a cohort navigator who will work closely with research staff and MSIR staff to facilitate participant recruitment. Research staff receive extensive training in obtaining informed consent and undertaking field-based data collection and survey administration,^67^ and adhere to standard operating procedures developed for fieldwork undertaken inside the MSIR. Recruitment commenced in September 2024 with final follow-ups to be completed by December 2027.

SIRX Study enrolment and survey completion will occur post-injection either inside the MSIR or in a research study van located outside the MSIR. Previous research demonstrates post-injection interviews and testing are feasible at the North Richmond MSIR.^53 68^ Researchers are trained to monitor for signs of heavy intoxication and reschedule interviews in line with current research standard operating protocols. Recruitment processes for SuperMIX and SIRX-R are detailed below.

#### SuperMIX cohort

A total of 1200 registered MSIR clients will be recruited into the SuperMIX Cohort for annual surveys and record linkage (including MSIR client records). This target recruitment sample consists of 360 participants already enrolled in SuperMIX and who report using the MSIR and approximately 840 new SuperMIX participants who are recruited directly from the MSIR. We will first consecutively invite all MSIR clients to participate in SuperMIX until the target sample size is reached. Participants recruited into SuperMIX will be reimbursed AUD$40 for baseline and follow-up surveys and AUD$10 for providing a blood-bio sample (total reimbursement AUD$50).

The SuperMIX Cohort will also provide a study control group of people who inject drugs who do not report using the MSIR (figure 1).

#### SIRX-R cohort

A total of 1800 participants will be recruited into the SIRX-R Cohort who will consent to record linkage (including MSIR client records) and complete a once-off cross-sectional survey. To streamline recruitment and reduce client burden, a SIRX Study data field will be added to the MSIR client database to identify clients who are (i) already enrolled in the study, (ii) are interested or can be approached about the study, or (iii) have declined study enrolment and should not be reapproached.

As outlined above, until SuperMIX Cohort recruitment targets are met, all eligible clients will first be invited to participate in the SuperMIX Cohort but where this is declined, clients will be invited to enrol in the SIRX-R Cohort. Once SuperMIX Cohort recruitment targets are met, all following recruitment will be for the SIRX-R Cohort.

All questionnaires will be administered using computers and tablets programmed using REDCap electronic data capture software hosted at the Burnet Institute.^69^

### Data sources

#### Self-report annual questionnaire data

The SuperMIX Cohort questionnaire collects information on sociodemographics, substance use and treatment, drug markets and purchasing, MSIR facility use and non-use, injecting-related harms, health and well-being including mental health (the PHQ-SADS – anxiety and depression)^70^ and social functioning (the SF8^71^ or EQ5-D) and the Personal Well-Being Index,^72^ health service use, stigma, and violence and criminal justice (table 1). The questionnaire for the SIRX-R Cohort will be a truncated version of the SuperMIX Cohort baseline questionnaire (questionnaires are available on request from the principal investigator).

**Table 1:** SIRX-R and SuperMIX data domains

Domain	Content example
Sociodemographics	Age, sex, gender identity, country of birth, ethnicity, indigenous identity, education and employment status, housing.
Substance use and treatment exposure	Drug type(s) used and injected, frequency of use and injecting, injecting initiation, current injecting behaviours, alcohol use, tobacco and e-cigarette use, substance use treatment.
Drug markets and purchasing	Cost and location of recent heroin and methamphetamine purchases.
MSIR visits	Frequency of visits, reasons for use, non-use, discontinuation of use.
MSIR on-site services	Frequency and type of on-site health and social service utilisation, provision and uptake of referrals to other health and social services
Injecting-related harms	Overdose history, recent opioid overdose, recent methamphetamine overdose, injecting-related injuries and infections, blood-borne viruses (including HIV/HCV treatment).
Health and well-being	General health conditions, women’s sexual and reproductive health, General Anxiety and Depression 7-Item Scale (GAD-7), reasons for health service access, EQ-5D, Patient Health Questionnaire (PHQ).
Other health service use	Use of other primary and specialist health services and reasons for use.
Stigma	Experience of stigma or discrimination related to drug use.
Violence and criminal justice	Violent victimisation, police contact, imprisonment history.
Trauma history	Experiences of child maltreatment and other psychological trauma.

MSIRMedically Supervised Injecting Room

#### Record linkage data

Record linkage to routinely collected administrative data sources to measure health service utilisation will occur over the study period, with participants also requested to consent for longer-term record linkage. Using the SuperMIX Cohort record linkage framework,^16^ participants will be asked to consent for linkage to databases capturing MSIR facility use, specialist and primary healthcare consultations, prescription medication dispensations, Victorian emergency and tertiary health service presentations, specialist drug treatment service contacts, criminal justice contacts, and death records. All databases are outlined in table 2. Linkage will be undertaken by accredited linkage authorities following approval from all data custodians. Linked data are deidentified and will be stored in a secure data storage and analysis environment such as the Sax Institute’s Secure Unified Research Environment (SURE).^73^ All analysis outputs are reviewed by the Sax Institute for compliance with data custodian requirements to ensure participant privacy is maintained.

**Table 2:** Administrative databases for linkage

Source database	Description	Key variables
MSIR Client Database*	Records of all client visits to the MSIR.	Visit date and time to determine number of visits per person.
MSIR Medical Record*	Records all on-site health and social services.	Medical comorbidity and on-site service provision.
Medicare Benefits Schedule (MBS)	Records of all primary healthcare services provided through government subsidised programme.	Service date, Medicare item number and description.
Pharmaceutical Benefits Scheme (PBS)	Records of all dispensations of medications available through the government-subsided programme.	Date of dispensation, medication type.
Victorian Admitted Episodes Database (VAED)	Records of all admissions and separations from Victorian hospitals.	Admission date and time, separation date and time, primary and secondary diagnoses, treatment procedures.
Victorian Emergency Minimum Dataset (VEMD)	Records of all presentations to Victorian hospital emergency departments.	Arrival date and time, separation date and time, mode of arrival, triage category, primary and secondary diagnoses, departure status.
Victorian Ambulance Clinical Information System (VACIS)	Records of all patient care events attended to by Ambulance Victoria.	Arrive date and time, location, patient clinical indicators, case information, primary incident assessment, transport.
Victorian Public Mental Health Database	Records of all episodes of care delivered by Victorian public mental health services including inpatient and community care.	Admission/contact date and time, separation date and time, crisis assessment, primary and secondary diagnoses, patient legal status.
Victorian Drug and Alcohol Collection (VADC)	Specialist drug and alcohol treatment service contacts in Victoria.	Treatment start and end date, service type, client substance use.
Law Enforcement Assistance Programme (LEAP)	Records of all contacts with Victoria Police.	Contact date and time, details of offence of reason for contact, outcome of police contact.
Corrections Victoria (CV)	All episodes of imprisonment in Victoria.	Prison reception and discharge dates.
National Death Index (NDI)	Mortality information for all deaths occurring in Australia.	Date and cause of death.

* These data will be used in exposure derivation.

MSIRMedically Supervised Injecting Room

### Measures

#### Outcomes

Derived from the legislated aims of the MSIR, the primary outcomes will be observed reductions in all-cause and drug-related mortality and ambulance attendances for non-fatal overdose. Secondary outcomes include reductions in drug-related hospitalisations such as injecting-related injuries and infections, and increased uptake of OAT (where indicated) and other non-acute health services.

#### Exposures

The primary exposure for the SIRX Study is time-varying frequency of MSIR use. Participants will be categorised as frequent (≥weekly) or infrequent (<weekly) users of the MSIR based on facility utilisation rates determined from MSIR client database records, which will vary across time as people change their frequency of MSIR use. For the SuperMIX Cohort, participants will also be able to be categorised as frequent (≥50% of their injections) or infrequent (<50% of their injections) users of the MSIR facility on the basis of self-report questions currently implemented in the SuperMIX survey, which collects information on the proportion of injections that took place in the MSIR in the past month.^74^ Analyses using this MSIR frequency threshold have been previously published.^38^ Sensitivity analyses will be considered to explore the MSIR use thresholds. Non-MSIR controls will be SuperMIX Cohort participants who indicate they have not used the MSIR, which may change over time.

### Statistical analyses

#### SIRX-R cohort

*Mortality*. We will undertake appropriate survival modelling (eg, Cox regression or parametric (accelerated failure time or proportional hazards)) to estimate the association between MSIR use and mortality, taking account of factors that might confound the association. In these models, MSIR use will be estimated as a time-varying exposure using person-period/episode split data. Prior use of the MSIR (before SIRX Study enrolment) will be considered when accounting for possible left truncation bias in these analyses. To provide more robust causal inference using data from the SIRX-R Cohort, we will also explore application of a case-time-control method to provide fixed effects estimation implicitly controlling for all time-invariant measured and unmeasured confounders (eg, prior overdose history, time since first injection, prior level of healthcare usage and treatment utilisation, general health at baseline, sex).^75^ This statistical model will be implemented via an exposure/outcome reversed conditional logistic regression analysis on discrete-time participant-period data representing the follow-up durations for each participant (eg, days), ending in either death or censorship.

*Ambulance-attended non-fatal overdose*. We will undertake generalised linear mixed modelling (GLMM, for example, Poisson (with bootstrapped standard errors) or negative binomial) on person-period data (ie, repeated ambulance-attended overdoses per participant measured periodically) to estimate the association between MSIR use and ambulance-attended non-fatal overdose. As stated for mortality analyses, we will implicitly control for all measured and unmeasured time-invariant confounders, a fixed-effects generalised linear modelling (Poisson or negative binomial) approach will be also explored using person-period data.

#### SuperMIX cohort

*Mortality and ambulance-attended non-fatal overdose*. To estimate the total causal effect (also referred to as the average causal treatment effect) of participant, MSIR use on mortality and ambulance-attended non-fatal overdose suitable causal inference statistical modelling (eg, marginal structural modelling^76^ (MSM) or sequential conditional mean modelling^77^ (SCMM)) will be undertaken on annual participant person-period data. In both these modelling methods and through different adjustment approaches (inverse probability weighting (IPW) for MSM and prior confounder/measure conditioning for SCMM possibly combined with IPWs to provide doubly robust estimation), time-independent and time-dependent confounding can be adjusted for to estimate total causal effects, without risk of introducing over-control bias (time-dependent confounding). We propose the application of MSM and/or SCMM given key differences between the methods in terms of levels of flexibility, bias and ease of implementation (ie, handling missing data and dropout, exposure and covariate interactions, estimation for continuous exposures, covariate/confounder history imbalance across exposed and unexposed treatment groups and precision; all favouring SCMM) and the ability to estimate direct longer term (*not total*) causal effects if required (only possible with MSM). Generalised linear modelling and generalised estimating equations will be used to implement MSMs and SCMMs respectively, with the appropriate distributional assumptions and link functions applied given specific outcome measurement (event-history modelling for morbidity and non-normal repeated measures estimating equations for ambulance-attended non-fatal overdose). Data-generating processes will be postulated using directed acyclic graphs, and these will inform the necessary structure of the statistical models to enable identification and estimation of total causal effects. Data-generating processes will also help inform the application of regression modelling.

#### Missing data treatment and attrition

Depending on the specific analysis being undertaken, a range of missing data strategies will be considered in terms of missing data and attrition. For analyses which entail GLMM, maximum likelihood estimation will be used, and this provides unbiased effect estimates using all participant observations assuming missingness due to attrition takes a missing-at-random (MAR) process (ie, missingness can be not ‘missing-completely-at-random’ (MCAR) and can depend on model covariates and the outcomes themselves at prior occasions (incl. random intercepts)). For MSMs, use of inverse probability treatment weights will incorporate a censoring weight component (based on covariates known to predict study drop-out). SCMMs produce unbiased estimates in the face of attrition when regression models include covariates known to predict study drop-out. Where there is considerable missing data on covariates in these analyses (eg, >10%), either multiple imputation or where possible (GLMM, linear mixed modelling (LMM)) full information maximum likelihood (FIML, implemented in Mplus) will be used for unbiased (assuming MAR) missing data treatment. Finally, in all survival analyses, we will perform sensitivity analyses to estimate the extent to which right-censoring in the data (including attrition) is informative with respect to the participant’s hazard of the outcome (eg, participants with a high hazard of non-fatal overdose may be more likely to be lost to follow-up). Non-informative censoring is a key assumption of survival analysis.

### Statistical power

Table 3 below details the approximate minimum detectable differences for mortality and non-fatal overdose analyses based on the expected distribution of participants in each cohort. Monte Carlo simulation modelling (using generalised linear mixed modelling (GLMM)) was used to estimate minimum detectable differences for non-fatal overdose and an exponential proportional hazards parametric survival model used for mortality. Baseline hazards (mortality: 1.1 per 100PY) and incidence rates (non-fatal overdose: 8.8 per 100PY) applying to each comparison were taken from current SuperMIX cohort data,^78^ as were the means and variance components used for the longitudinal GLMM simulations. All effect size estimations assumed 80% power and 5% significance. For Monte Carlo simulated analyses (n=300 replications), simulations were based on three annual outcome measurements and expected cohort attrition (SuperMIX analyses) of 30% in year 1 and 25% thereafter.

**Table 3:** Minimum detectable effect sizes for outcomes

SIRX study	Comparison	Mortality (HR)	Non-fatal OD (IRR)
SIRX-R Cohort	Frequent (weekly) vs infrequent (<weekly) use	0.42	0.69
SuperMIX Cohort	Frequent (≥50% all injections) vs no use	0.3	0.58
SuperMIX Cohort	Infrequent (<50% injections) vs no use	0.37	0.64

IRRincidence rate ratioODoverdoseSIRX-RSupervised Injecting Room Registration CohortSuperMIXMelbourne Injecting Drug User Cohort

### Economic evaluation

Cost data will be available to enable economic modelling. Health economic outcomes will be considered from a government perspective, compared across the categories of MSIR exposure after weighting for cohort size. The main outcomes will be (1) the difference in total annual costs; (2) the cost per life saved; and (3) the cost per quality-adjusted life year (QALY) gained. Total costs will include costs associated with MSIR use (calculated from financial documentation and budgets over time), costs of ambulance callouts for overdoses (available on Ambulance Victoria website), healthcare costs (matching linked healthcare usage data with corresponding MBS/PBS codes, in particular costs associated with managing blood-borne virus or injecting related injuries), and OAT treatment costs. QALY gains will be estimated based on additional OAT uptake, treatment of comorbidities (eg, person-years lived with hepatitis C), changes in employment, and deaths averted that are attributable to the MSIR. Cost per QALY gained outcomes will enable benchmarking of the MSIR against other health interventions.

## Patient and public involvement

The SIRX Study was designed in partnership with key stakeholders including Harm Reduction Victoria, the representative body for people who use and inject drugs in Victoria; North Richmond Community Health, the primary health service operating the Melbourne MSIR; cohealth, a not-for-profit community health organisation providing health services including services and support for alcohol and other drugs; and the Victorian State Government. These stakeholders, along with study investigators, contribute to ongoing oversight of the study via their involvement in the SIRX Study Research Advisory group.

## Discussion

The SIRX Study has been designed to provide new evidence on the effects of SIFs in reducing overdose deaths and drug-related harms within the Australian context. Previous research has highlighted the benefits of the Melbourne MSIR but is limited by short evaluation timeframes, reliance on ecological data, or the absence of temporality to control for confounding and determine causation.^38 50 58^ Drawing on the cohort methodology used to evaluate the Insite SIF in Canada,^66^ our study aims to generate quantitative evidence of the impact of the MSIR including its model of on-site health and social service delivery, overcoming the limitations described above. The results can be used to inform decisions about the value of SIFs in general, and the specific value of a model embedding the SIF within a range of on-site health and social services.

### Limitations

The SIRX study, while valuable in measuring the effects of Melbourne’s MSIRs in reducing drug-related harm, is subject to several limitations. Self-report data may be subject to responses biases, including recall and socially desirable responses. The SuperMIX Cohort component may be subject to lost-to-follow-up; previous work demonstrated stable attrition in the SuperMIX Cohort with higher attrition among individuals with greater risk profiles.^79^ However, using linked data for primary outcomes mitigates the risk of such biases. Despite the large projected sample size, the expected effect estimates for mortality remain relatively imprecise in contrast to the effects on non-fatal overdose. As with all observational studies, confounding may impact the observed associations, but casual inference methods are being applied to minimise this. Finally, selection bias may mean the study is not representative of all Melbourne MSIR clients and findings may not be generalisable to other SIFs. However, the use of comprehensive time-dependent data collected across a range of individual and health-related factors, combined with the use of casual inference methods, means the SIRX Study will generate strong evidence on the causal effects of SIFs.

## Ethics and dissemination

### Ethics approval

SuperMIX Study (599/21) and SIRX-R Study (71/23) ethics approvals were obtained from Alfred Hospital Research Ethics Committee. Participants will be assessed for capacity to provide informed consent following a detailed explanation of the study. Participants are informed of their right to withdraw from the study at any time and that withdrawing does not impact their access to services.

### Results dissemination

Aggregated research results will be disseminated via presentations at national and international scientific conferences and publications in peer-reviewed journals. Local-level reports and outputs will be distributed to key study stakeholders and policymakers. Summary findings for participants will be displayed in relevant services and the study van, via accessible outputs (eg, short infographics summaries).
