# Supplementary material for: Supervised Injecting Room Cohort Study (SIRX): study protocol
Source: BMJ Open. 2025 Feb 11;15(2):e091337. doi: 10.1136/bmjopen-2024-091337 (PMC11815420; doi:10.1136/bmjopen-2024-091337)
Supplement: Supplementary file 1 [file bmjopen-2024-091337.reviewer_comments.pdf]

PEER REVIEW HISTORY

BMJ Open publishes all reviews undertaken for accepted manuscripts. Reviewers are asked to complete a checklist review form and are provided with free text boxes to elaborate on their assessment. These free text comments are reproduced below.

ARTICLE DETAILS

Title (Provisional)

Supervised injecting room cohort study (SIRX): study protocol

Authors

Stewart, Ashleigh Cara; Hickman, Matthew; Agius, Paul A.; Scott, Nick; Stone, Jack; Roxburgh, Amanda; O'Keefe, Daniel; Higgs, Peter; Kerr, Thomas; Stoové, Mark A; Thompson, Alexander; Crawford, S; Norman, Josephine; Vella-Horne, Dylan; Lloyd, Zachary; Clark, Nico; Maher, Lisa; Dietze, Paul

---

VERSION 1 - REVIEW

**Reviewer**	**1**
**Name**	**Urbanik, Marta-Marika**
**Affiliation**	**University of Alberta, Sociology**
**Date**	**03-Oct-2024**
**COI**	**None**

Review of "Supervised injecting room cohort study (SIRX): study protocol." BMJ.

Thank you for the opportunity to review this proposal. The author(s) are correct in highlighting the limited solid and longitudinal research exploring the effectiveness of SIRs, especially in a context beyond Vancouver's Insite—where most of this type of research is based. Using a quasi-experimental longitudinal/cohort design, this protocol paper aims to assess the effectiveness of a SIF in Melbourne in reducing drug-related harms and delivering additional services to vulnerable people who use drugs. The study's purported emphasis on examining whether/how SIR exposure levels (especially frequency of us) affect various aims of SIRs is especially commendable and will be a significant contribution to the literature.

This manuscript provides an empirically driven rationale for comparing all-time and intermittent SIF users' health records. In its current form, minor revisions will benefit the proposal, which are presented below. I recognize word limits may constrain the implementation of some of these suggestions, so I recommend that the authors determine what changes to prioritize.

A key concern that emerges is how the study question/basis/justification is presented. The proposal routinely emphasizes that the study's purpose is to "provide the strongest possible evidence of the effectiveness of the MSRI" and to "provide further evidence…" This reads as though the study is intended to ONLY provide scientific evidence that the MSRI is effective across a range of factors and may suggest to readers that the study proposal (and thus the study) is designed in a biased way, intended to solely support the MSRI's benefits. What if the author(s) uncover limited MSRI benefit? What if the author(s) identify areas where the MSRI may not be meeting its goal? Will these outcomes be shared? This seems like the authors have their purported 'evidence' and conclusion before the onset of the study. The author(s) are encouraged to present and pursue this study with academic and empirical openness, thus perhaps rephrasing the study's objectives/questions to determine whether, to what extent, and why (or why not) the MSRI is effective on the range of outcomes they outline. Even if there is existing evidence and thus the author(s) believe they need to provide "new evidence," this does not preclude initiating the study with an open mind; this is an improved technique and research method—which I commend the author(s) for—thus the proposal to examine the effects needs not to be qualified with the prior study's data. The need to be, and present as, scientifically sound and objective is especially critical given the extremely politicized nature of SIRs and often broad pushback to them and other forms of critical harm reduction services.

I am curious about the selection of using the SRI for over/under 50% of injections. What was the basis for this selection? Is this likely to have the best reflection of PWID experiences locally? Can this be, pre or post, broken down into more segments, such as 70%, 50%, 30%, etc? I ask because the 50% threshold will be grouping likely very different types of PWID together, potentially masking critical differences in their experiences. Someone who uses the site say, 4/10 times they consume and someone who uses it less than 1/10 times they consume—say, when its convenient for them—are likely going to have different outcomes/experiences/and critically, be experiencing different types of marginalization (or access barriers).

The current research protocol (and future full manuscripts emerging from this protocol) will benefit from weaving the current local drug scene of Melbourne into the objective of the paper. Are there additional reasons to conduct this study beyond measuring the effectiveness of SIF/MSIR? Is there any literature on how many people are there (approx) who use drugs in public spaces in Melbourne? Where do people who use drugs seek support from? Are their needs met, and to what extent?

Other suggestions:

- "Neighbourhood amenity" can this be rephrased so that more readers can understand what amenity refers to this in respect? In the North American context it is typically understood as a service/benefit.

- Perhaps find a more accessible word to replace “equipoise,” especially for international readers.

- “including drug related crime and avoidable healthcare costs”- suggest including criminalization which may be, but is not necessarily related to harm, as broadly conceived given its unequal application to marginalized persons and PWID (e.g., loitering). This also exacerbates harm for marginalized persons and PWID.

- “undermining access to supportive services, such as OAT”- suggest including harm reduction here too, which has been established across the literature but may not be automatically associated with ‘supportive services.’

- “suboptimal program coverage is common in Australia”- please re-word as this is awkwardly and unclearly written.

- The authors could explain in detail the debate surrounding the opening of the second SIF in Melbourne to offer more compelling and clearer context. On p. 6, it reads “In 2024, the commitment to establish the second facility was withdrawn.” Three lines later, it states: “The withdrawal of the commitment to establish a second facility highlights the precarious situation of SIFs and the need for robust evidence on the effectiveness of SIFs, particularly in light of suggestions by the Victorian opposition that they would close the MSIR if elected could better.” This suggests other reasons, perhaps more politically motivated, that deter the establishment of SIFs. To strengthen the manuscript, the authors could contextualize (1) where the new SIF/MSIR was intended to be located, (2) whether and why there was resistance from local stakeholders, such as business owners or residents in the area where the facility was planned to operate. Contextualizing this manuscript (even in its research protocol form) within the broader context of Melbourne's drug scene would help reveal other factors and reasons necessitating this research. Given word limitations, this could be a short explanation OR the authors could cite newsmedia/other reports so interested readers can pursue this on their own.

- The authors use medical jargon and acronyms (e.g., SIRX, MSIR, SuperMix) that appear similar. This could confuse readers outside of the medical field. Perhaps consider replacing them with other terms that meet the needs of a broader general audience.

- In the following sentence, “Using the SuperMIX Cohort record linkage framework, participants’ will be asked to consent for linkage to databases capturing … (p. 10)” – please remove the apostrophe after ‘participants’.

- The strength of this manuscript is its comprehensive data analysis approach that considers and addresses possible limitations of the study. The authors clearly demonstrate how the collected data will be quantified and analyzed, accounting for the possibility of confounding variables attributed to selection bias, attrition rate, etc. However, are there any other potential confounding variables that warrant examination? For instance, participants’ gender

identity is included in Table 1. Would the exclusion of sexual orientation increase the effect of confounding variables?

- Please reframe the abstract. The paper reads better and becomes more coherent towards the end. The beginning (especially the abstract) needs some rework.

- The growth in opioid-related deaths in Australia since 2001 comes a bit late; consider moving it up to further underscore the need for the study

- It is advisable to include a sentence and citations demonstrating why there may be push-back to SIR, especially concerns with respect to perceptions of increasing crime, disorder, decreasing property values, changing government mandates and priorities (e.g., from harm reduction to 'recovery first') etc. See for example, Greene et al. "It's just not the same" in IJDP or Livingston's 2021 "Supervised consumption sites and crime" in Harm Reduction Journal.

- A careful copy-edit is needed, a few errors noted. For example: line 5 right before the methods (missing an and?), line 26 under SUPERMIX (missing a 'who'?)

- When examining reasons for not using the SIR, I encourage the authors to probe these barriers/preferences, including recognizing and documenting how many/why individuals may have been banned from doing so and for how long (this need not be reflected in this proposal but is a point to consider going forward as additional research on this topic is necessary).

- "peak body" seems like odd phrasing; perhaps reconsider changing it.

**Reviewer**	**2**
**Name**	**Otiashvili, David**
**Affiliation**	**Addiction Research Center, Alternative Georgia**
**Date**	**07-Oct-2024**
**COI**	**None**

Overall Evaluation

This study protocol addresses an important gap in the evaluation of SIFs and has the potential to generate significant insights for harm reduction services. While the study protocol is largely comprehensive, there are areas where further clarity and elaboration are needed to enhance its transparency and replicability, particularly in the methods section.

Specific comments

Study setting:

• The manuscript provides a comprehensive description of the study setting (Melbourne, Australia, and the MSIR). This is well done.

Eligibility criteria:

• The eligibility criteria for MSIR clients are well defined. However, a more detailed description of the inclusion/exclusion criteria for the SuperMIX cohort could be beneficial.

Exposure to the MSIR:

• More detail on the different categories of MSIR use (frequent vs. infrequent) and how this data will be collected would be useful for replication.

Outcomes:

• Primary and secondary outcomes are clearly stated, focusing on mortality, non-fatal overdose, and health service use. Further justification for the selection of secondary outcomes (e.g., public syringe sharing, hepatitis C treatment) would strengthen the rationale.

Sample size and recruitment:

• Sample size estimates are clearly explained, but the manuscript could benefit from more detail on recruitment strategies to ensure adequate enrolment in the two cohorts (SuperMIX and SIRX-R).

Data collection methods:

• The protocol outlines the plan for collecting data through behavioural surveys and administrative record linkage. A more thorough description of how data quality will be ensured (e.g., training for data collectors) would be useful.

Statistical methods:

• The statistical methods section is well developed, with appropriate consideration of confounding factors using causal inference methods. The use of time-varying exposures is well justified. However, more detail on the handling of missing data would strengthen this section.

Harms:

• The manuscript briefly mentions potential harms associated with the study but lacks a clear plan for how adverse events will be recorded and managed.

Consent process:

• The protocol outlines that informed consent will be obtained, but the process for obtaining it could benefit from further elaboration. Specifically, it would be useful to describe who will obtain consent, how they will be trained to ensure consistency, and whether participants will be re-consented if there are any protocol changes. Additionally, the manuscript should

provide details on whether any additional consent will be required for the use of participant data in ancillary studies, should those arise.

Confidentiality:

• The manuscript mentions the use of data linkage for administrative records, which raises confidentiality concerns. It would be helpful to explain the measures in place to protect the personal information of participants, especially with respect to the handling of sensitive health data before, during, and after the study. Clarifying how identifiable information will be shared or stored, and what steps will be taken to anonymize data for analysis, would further enhance the protocol's commitment to protecting participant privacy.

Ethics and dissemination:

• The ethics approval process is clearly described. Plans for dissemination are outlined, but more detail on how the authors plan to engage with study participants and provide feedback on study results would be beneficial.

**Reviewer**	**3**
**Name**	**Cousien, Anthony**
**Affiliation**	**Sorbonne-Paris**
**Date**	**16-Oct-2024**
**COI**	**None**

The protocol presents a cohort study with a high interest for research on harm reduction among people who inject drugs. The protocol is clear and well describe, and I only have a concern regarding the statistical analysis.

Detailled comments:

Page 6, lines 7-9: "Despite this evidence base, the evaluation of SIFs remains challenging with researchers determining randomised control trials to be unethical for evaluating the effect of SIFs, particularly in the absence of clinical equipoise when intervening in the event of an overdose." There are also feasibility and implementations problems for such RCTs.

Methods : the dates (start and end) of the study are missing, they are only presented on Figure 1 and should be in the text.

Page 9, line 6: "Previous experience indicates high response rates using these methods." What was the range ?

Page 12-13, paragraph "Mortality and ambulance-attended non-fatal overdose.": The statistical analysis plan is poorly described. Why did the authors consider at least two

methods (MSM and SCMM)? As they mentioned “e.g.” before them, I assume there are other candidates? Would it not be possible to choose one of them know?

Also, page 12, lines 59-60 “under certain assumptions and conditions”: what are these assumptions? Would the data be in verify them?

Page 13: the minimum detectable effect for mortality HR would be 0.3 to 0.42. It’s seems very high. Have the authors looked for published studies supporting the possibility to reach this level?

Similarly, some data could be used to put in perspective the minimum detectable effect on non-fatal OD (e.g. https://pubmed.ncbi.nlm.nih.gov/35690956/)

Page 13-14, paragraph “Economic evaluation”. Do the authors plan to use modelling to project long-term effect? As they plan to account for long term diseases (e.g. hepatitis C)?

VERSION 1 - AUTHOR RESPONSE

Reviewer: 1

Overall, this reviewer has provided several suggestions for amending the manuscript to include further detail related to supervised injecting rooms and specifically in the Melbourne context. We have now amended the manuscript in serval places to provide details of the model for the Melbourne MSIR in being co-located with a community health service and providing integrated health and social support services. These edits have largely been made in the abstract background and within the section titled ‘The Melbourne Medically Supervised Injecting Room (MSIR)’. We have included some examples below amendments, with all amendments included as tracked changes in the manuscript document.

“Supervised injecting facilities (SIFs) are designed to reduce the harms associated with injecting drug use and improve access to health and support services for people who need them. The Supervised Injecting Room Cohort Study (SIRX) aims to provide evidence of the effects, including cost-effectiveness, of a SIF embedded within a community health service, the Melbourne Medically Supervised Injecting Room (MSIR), which has a range of integrated harm reduction, health and social support services on-site.” (page 3)

“SIFs provide an emergency response in the event of drug overdose, facilitate referrals to other health and social services providers, and sometimes also provide a range of on-site services. While referrals from SIFs have been shown to result in the uptake of services[26], people who use SIFs have also demonstrated a preference to receive care on-site, due to the relationship with SIF staff, and negative experiences with mainstream health services.[27]” (page 6)

“Based on consultations with people injecting drugs in the local area, the Melbourne MSIR was designed to meet their needs as a “one-stop shop”, incorporating an extensive range of on-site health and social services,[52] including BBV testing and treatment,[53, 54] OAT,[55] oral health care, housing support, wound care, mental health support, legal assistance, food, and primary care, as well as referral to other services when needed. The design of each of the health and social services was optimised to be responsive to the needs of the people who inject drugs, by offering simplified

treatment pathways, incorporating increased flexibility in service delivery and utilising a trauma-informed approach. This resulted in a substantial uptake in on-site services, including 387 treatment initiations for Hepatitis C, and 1096 initiations of OAT,[56] in some ways distinguishing the MSIR from other SIFs." (page 7)

1. A key concern that emerges is how the study question/basis/justification is presented. The proposal routinely emphasizes that the study's purpose is to "provide the strongest possible evidence of the effectiveness of the MSRI" and to "provide further evidence…" This reads as though the study is intended to ONLY provide scientific evidence that the MSRI is effective across a range of factors and may suggest to readers that the study proposal (and thus the study) is designed in a biased way, intended to solely support the MSRI's benefits. What if the author(s) uncover limited MSRI benefit? What if the author(s) identify areas where the MSRI may not be meeting its goal? Will these outcomes be shared? This seems like the authors have their purported 'evidence' and conclusion before the onset of the study. The author(s) are encouraged to present and pursue this study with academic and empirical openness, thus perhaps rephrasing the study's objectives/questions to determine whether, to what extent, and why (or why not) the MSRI is effective on the range of outcomes they outline. Even if there is existing evidence and thus the author(s) believe they need to provide "new evidence," this does not preclude initiating the study with an open mind; this is an improved technique and research method—which I commend the author(s) for—thus the proposal to examine the effects needs not to be qualified with the prior study's data. The need to be, and present as, scientifically sound and objective is especially critical given the extremely politicized nature of SIRs and often broad pushback to them and other forms of critical harm reduction services.

It was never our intention to present only partial evidence of the effectiveness of the Melbourne SIF. We agree with the reviewer suggestions and have amended language throughout the manuscript to present unbiased aims and objectives and the study aims now refer to generating evidence of the 'effects' of the Melbourne MSIR.

"The Supervised Injecting Room Cohort Study (SIRX) aims to provide evidence of the effects, including cost-effectiveness, of a SIF embedded within a community health service, the Melbourne Medically Supervised Injecting Room (MSIR), which has a range of integrated harm reduction, health and social support services on-site". (page 3)

"SIRX aims to provide further evidence of the effects, …" (Page 9)

"The SIRX Study has been designed to provide new evidence on the effects of SIFs …" (page 16)

2. I am curious about the selection of using the SRI for over/under 50% of injections. What was the basis for this selection? Is this likely to have the best reflection of PWID experiences locally? Can this be, pre or post, broken down into more segments, such as 70%, 50%, 30%, etc? I ask because the 50% threshold will be grouping likely very different types of PWID together, potentially masking critical differences in their experiences. Someone who uses the site say, 4/10 times they consume and someone who uses it less than 1/10 times they consume—say, when its convenient for them—are likely going to have different outcomes/experiences/and critically, be experiencing different types of marginalization (or access barriers).

This choice was a pragmatic decision based on a previous analysis exploring frequency of MSIR use among participants in the SuperMIX cohort study (van Den Boom et al., 2021). We have included a statement referring to this publication as rationale for this frequency of use threshold and we can explore this threshold in sensitivity analysis and have noted this in the manuscript.

"Analyses using this MSIR frequency threshold has been previously published.[38] Sensitivity analyses will be considered to explore the MSIR use thresholds." (page 13).

Van Den Boom, W., del Mar Quiroga, M., Fetene, D. M., Agius, P. A., Higgs, P. G., Maher, L., Hickman, M., Stoové, M. A., & Dietze, P. M. (2021). The Melbourne Safe Injecting Room Attracted People Most in Need of Its Service. American Journal of Preventive Medicine, 61(2), 217–224. https://doi.org/10.1016/j.amepre.2021.02.018

3. The current research protocol (and future full manuscripts emerging from this protocol) will benefit from weaving the current local drug scene of Melbourne into the objective of the paper. Are there additional reasons to conduct this study beyond measuring the effectiveness of SIF/MSIR? Is there any literature on how many people are there (approx.) who use drugs in public spaces in Melbourne? Where do people who use drugs seek support from? Are their needs met, and to what extent?

Thank you for these comments. Although there are no area-specific data on the number of people who inject drugs in public we have elaborated on the visible nature of the North Richmond drug market from previous publications. We have included detail on the nature of the services specifically available in the MSIR itself to highlight the way the model operates and how this model was developed in consultation with people who inject drugs to meet their needs. We have also expanded on some of the measures relevant to this model of service delivery in the methods.

"Prior to the establishment of the Melbourne MSIR, local drug market activity in the North Richmond area was characterised by highly visible public injecting, injecting-related litter and high rates of fatal and non-fatal drug overdoses.[49-51]" (page 7)

4. "Neighbourhood amenity" can this be rephrased so that more readers can understand what amenity refers to this in respect? In the North American context it is typically understood as a service/benefit.

We have retained the phrase 'neighbourhood amenity' as this was a legislated aim of the Melbourne MSIR and thus referred in the MSIR review report. We have included clarification for this in the manuscript for international readership and cited a recent publication exploring 'public amenity' in the context of the Melbourne MSIR and how this was referred in print media.

"…. (this was not defined but typically relates to public injecting, public overdose, discarded injecting equipment, and perceived safety of the surrounding environment[57]);…" (page 8).

5. Perhaps find a more accessible word to replace "equipoise," especially for international readers.

We believe 'clinical equipoise' is a suitable term for the scientific community but have added in brackets *"(uncertainty over evidential strength for an intervention)" (page 7).*

6. “including drug related crime and avoidable healthcare costs”- suggest including criminalization which may be, but is not necessarily related to harm, as broadly conceived given its unequal application to marginalized persons and PWID (e.g., loitering). This also exacerbates harm for marginalized persons and PWID.

We have amended the manuscript to include a statement linking the criminalisation of injecting drug use to its health and social impacts.

“Injecting drug use is also connected to wider social and economic harms, including drug-related crime[7] and avoidable healthcare costs[8,9] many of which are related to current drug policy and the *criminalisation of illicit drugs.[10]” (page 6).*

7. “undermining access to supportive services, such as OAT”- suggest including harm reduction here too, which has been established across the literature but may not be automatically associated with ‘supportive services.’

We have amended based on this reviewer’s recommendation.

“Stigma and discrimination, in particular, contributes to suboptimal health care, undermining access to supportive and harm reduction services, such as opioid agonist therapy (OAT).” (page 6).

8. “suboptimal program coverage is common in Australia”- please re-word as this is awkwardly and unclearly written.

We have amended this sentence to improve readability.

“… but program coverage of these interventions is variable in Australia and may be insufficient to reduce drug-related harms.[24]*” (page 6).*

9. The authors could explain in detail the debate surrounding the opening of the second SIF in Melbourne to offer more compelling and clearer context. On p. 6, it reads “In 2024, the commitment to establish the second facility was withdrawn.” Three lines later, it states: “The withdrawal of the commitment to establish a second facility highlights the precarious situation of SIFs and the need for robust evidence on the effectiveness of SIFs, particularly in light of suggestions by the Victorian opposition that they would close the MSIR if elected could better.” This suggests other reasons, perhaps more politically motivated, that deter the establishment of SIFs. To strengthen the manuscript, the authors could contextualize (1) where the new SIF/MSIR was intended to be located, (2) whether and why there was resistance from local stakeholders, such as business owners or residents in the area where the facility was planned to operate. Contextualizing this manuscript (even in its research protocol form) within the broader context of Melbourne's drug scene would help reveal other factors and reasons necessitating this research. Given word limitations, this could be a short explanation OR the authors could cite news media/other reports so interested readers can pursue this on their own.

Keeping word limitations in mind, we have amended to include a statement providing context for the withdrawal of the second Melbourne MSIR.

“In 2024 following strong resistance from business owners and residents, driven by ongoing negative media and concerns about potential increases in crime and impacts on property values,[60-62] as well as

the failure to establish a suitable location,[63] the commitment to establish the second facility was withdrawn.[64]" (page 8)

10. The authors use medical jargon and acronyms (e.g., SIRX, MSIR, SuperMix) that appear similar. This could confuse readers outside of the medical field. Perhaps consider replacing them with other terms that meet the needs of a broader general audience.

All acronyms are defined in full and then subsequently used throughout the manuscript. We have chosen not to replace acronyms but will take editorial advice.

11. In the following sentence, "Using the SuperMIX Cohort record linkage framework, participants' will be asked to consent for linkage to databases capturing … (p. 10)" – please remove the apostrophe after 'participants'.

We have removed the apostrophe as recommended.

12. The strength of this manuscript is its comprehensive data analysis approach that considers and addresses possible limitations of the study. The authors clearly demonstrate how the collected data will be quantified and analyzed, accounting for the possibility of confounding variables attributed to selection bias, attrition rate, etc. However, are there any other potential confounding variables that warrant examination? For instance, participants' gender identity is included in Table 1. Would the exclusion of sexual orientation increase the effect of confounding variables?

Table 1 provides an overview of data domains and information collected in the SuperMIX and SIRX-R surveys but is not an exhaustive list. The reviewer makes an important point and to highlight the range of variables that are collected and provide clarity on this being a content example, we have amended the table column header to content example (page 11). We have noted that questionnaires are available on request and have therefore not amended to include further information.

13. Please reframe the abstract. The paper reads better and becomes more coherent towards the end. The beginning (especially the abstract) needs some rework.

We have amended the abstract for clarity and removed the conclusion as per editorial advice. Amendments are visible as tracked changes within the manuscript document (page 3).

14. The growth in opioid-related deaths in Australia since 2001 comes a bit late; consider moving it up to further underscore the need for the study

We have amended the manuscript as per this suggestion from the reviewer. The statement on the doubling of opioid-related deaths in Australia is now made in the first paragraph.

"Regular injecting drug use is associated with a wide range of adverse health outcomes including drug overdose deaths, which have steadily increased in the last two decades in Australia and globally.[1, 2] In particular, opioid-related deaths in Australia have doubled since 2001.[3]" (page 6)

15. It is advisable to include a sentence and citations demonstrating why there may be push-back to SIR, especially concerns with respect to perceptions of increasing crime, disorder, decreasing property values, changing government mandates and priorities (e.g., from harm reduction to 'recovery first') etc. See for example, Greene et al. "It's just not the same" in IJDP or Livingston's 2021 "Supervised consumption sites and crime" in Harm Reduction Journal.

As suggested, we have included this context for resistance towards SIFs.

"In 2024 following strong resistance from business owners and residents, driven by ongoing negative media and concerns about potential increases in crime and impacts on property values,[60-62] *as well as the failure to establish a suitable location,*[63] *the commitment to establish the second facility was withdrawn.*[64]*" (page 8)*

16. A careful copy-edit is needed, a few errors noted. For example: line 5 right before the methods (missing an and?), line 26 under SUPERMIX (missing a 'who'?)

We have amended these errors as requested and undertaken a careful review of the manuscript.

17. When examining reasons for not using the SIR, I encourage the authors to probe these barriers/preferences, including recognizing and documenting how many/why individuals may have been banned from doing so and for how long (this need not be reflected in this proposal but is a point to consider going forward as additional research on this topic is necessary).

Thank you for this suggestion, we do collect this information, which is detailed in Table 1 'MSIR visits – reasons for use, non-use, discontinuation of use'. As noted above in our response to comment 14, this table provides an example of content collected and is not an exhaustive list of all survey questions.

18. "peak body" seems like odd phrasing; perhaps reconsider changing it.

We have amended this phrasing to 'representative body' (page 16).

Reviewer: 2

19. The eligibility criteria for MSIR clients are well defined. However, a more detailed description of the inclusion/exclusion criteria for the SuperMIX cohort could be beneficial.

We have highlighted that eligibility criteria for SuperMIX are aligned to criteria outlined for SIRX and cited the SuperMIX cohort profile (page 10).

20. More detail on the different categories of MSIR use (frequent vs. infrequent) and how this data will be collected would be useful for replication.

The manuscript outlines that frequency of MSIR use is based on i) facility utilisation rates drawn from linkage to MSIR client database records, or ii) based on responses to self-report questions collected via the SuperMIX survey. We have amended the manuscript to outline that self-report data relates to *"the proportion of injections that took place in the MSIR in the past month" (page 13).*

21. Primary and secondary outcomes are clearly stated, focusing on mortality, non-fatal overdose, and health service use. Further justification for the selection of secondary outcomes (e.g., public syringe sharing, hepatitis C treatment) would strengthen the rationale.

We thank the reviewer for this suggestion and have amended the manuscript in several places to include justification for secondary outcomes by detailing the on-site services provided as part of this Melbourne MSIR model which is embedded within a community health service. Please see examples of these amendments provided in the overall comment at the beginning of this document. All amendments are tracked within the manuscript document.

22. Sample size estimates are clearly explained, but the manuscript could benefit from more detail on recruitment strategies to ensure adequate enrolment in the two cohorts (SuperMIX and SIRX-R).

The manuscript details the role of a 'cohort navigator' who works closely with research staff and MSIR staff to facilitate participant recruitment. We have amended the manuscript to include a statement about research staff receiving extensive training in field-based data collection and survey administration and have cited the SuperMIX baseline paper that details this further.

"Recruitment inside the MSIR will be led by a cohort navigator who will work closely with research staff and MSIR staff to facilitate participant recruitment. Research staff receive extensive training in obtaining informed consent and undertaking field-based data collection and survey administration[67] and follow standard operating procedures developed for fieldwork undertaken inside the MSIR." (page 10)

23. The protocol outlines the plan for collecting data through behavioural surveys and administrative record linkage. A more thorough description of how data quality will be ensured (e.g., training for data collectors) would be useful.

Please refer to the above response and amendment that outlines research staff receiving extensive training in field-based data collection and survey administration.

24. The statistical methods section is well developed, with appropriate consideration of confounding factors using causal inference methods. The use of time-varying exposures is well justified. However, more detail on the handling of missing data would strengthen this section.

We now include a detailed missing data treatment and attrition section in the manuscript which outlines approaches we will consider applying in dealing with missing exposure and outcome data where encountered.

"Missing data treatment and attrition

Depending on the specific analysis being undertaken, a range of missing data strategies will be considered in terms of missing data and attrition. For analyses which entail GLMM, maximum likelihood estimation will be used, and this provides unbiased effect estimates using all participant observations assuming missingness due to attrition takes a missing-at-random (MAR) process (i.e. missingness can be not 'missing-completely-at-random (MCAR) and can depend on model covariates

and the outcomes themselves at prior occasions (incl. random intercepts). For MSMs, use of inverse probability treatment weights will incorporate a censoring weight component (based on covariates known to predict study drop-out). SCMMs produce unbiased estimates in the face of attrition when regression models include covariates known to predict study drop-out. Where there is considerable missing data on covariates in these analyses (e.g. >10%), either multiple imputation or where possible (GLMM, linear mixed modelling (LMM)) full information maximum likelihood (FIML, implemented in Mplus) will be used for unbiased (assuming MAR) missing data treatment. Finally, in all survival analyses, we will perform sensitivity analyses to estimate the extent to which right-censoring in the data (including attrition) is informative with respect to the participant's hazard of the outcome (e.g. participants with a high hazard of non-fatal overdose may be more likely to be lost to follow up). Non-informative censoring is a key assumption of survival analysis." (page 15)

25. The manuscript briefly mentions potential harms associated with the study but lacks a clear plan for how adverse events will be recorded and managed.

It is unclear what types of 'potential harms' the reviewer is referring. Study limitations are detailed in the 'limitations' section of the manuscript (page 17), with approaches to mitigating such limitations outlined. We also note that concerns relating to post-injection interviewing are detailed (page 10), with reference to previous research that demonstrates the feasibility of this, and that staff are trained to monitor signs of intoxication and to reschedule interviews where needed. We also note the above amendment indicating that research staff undertake extensive training for field-based data collection and follow a standard operating procedures for fieldwork undertaken inside the MSIR.

26. The protocol outlines that informed consent will be obtained, but the process for obtaining it could benefit from further elaboration. Specifically, it would be useful to describe who will obtain consent, how they will be trained to ensure consistency, and whether participants will be re-consented if there are any protocol changes. Additionally, the manuscript should provide details on whether any additional consent will be required for the use of participant data in ancillary studies, should those arise.

We have amended the manuscript to include a statement about research staff being trained in obtaining informed consent. See comment above.

SuperMIX participants can be reconsented in the case of protocol amendments, but SIRX-R participants complete a once off survey and do not provide contact information, thus cannot be reconsented. Participant consent includes a statement for unspecified consent processes, which details where participants consent to their data (incl., blood samples) being used in other ethically approved research led by SuperMIX investigators. We have amended the manuscript to include a statement on this.

"MSIR clients will be eligible for the SIRX Study if they attend and use the MSIR in the six months prior to study contact and consent to an interview and record linkage. Participants also provide unspecified consent for their data to be used in other ethically approved studies involving project investigators." (page 9)

27. The manuscript mentions the use of data linkage for administrative records, which raises confidentiality concerns. It would be helpful to explain the measures in place to protect the

personal information of participants, especially with respect to the handling of sensitive health data before, during, and after the study. Clarifying how identifiable information will be shared or stored, and what steps will be taken to anonymize data for analysis, would further enhance the protocol's commitment to protecting participant privacy.

We have amended the manuscript to include a statement about the data output curation process within the secure research environment where data are stored. This process ensures compliance for non-identifiable data and participant privacy.

"Linked data are deidentified and will be stored in a secure data storage and analysis environment such as the Sax Institute's Secure Unified Research Environment (SURE).[73] 16 All analysis outputs are reviewed by the Sax Institute for compliance with data custodian requirements to ensure participant privacy is maintained." (page 12).

28. The ethics approval process is clearly described. Plans for dissemination are outlined, but more detail on how the authors plan to engage with study participants and provide feedback on study results would be beneficial.

We have amended the manuscript and included further detail in 'ethics and dissemination'.

"Summary findings via accessible outputs (e.g., short infographic summaries) for participants will be displayed in relevant services including the Melbourne MSIR and the study van, and will be distributed via Harm Reduction Victoria." (page 3)

Reviewer: 3

29. Page 6, lines 7-9: "Despite this evidence base, the evaluation of SIFs remains challenging with researchers determining randomised control trials to be unethical for evaluating the effect of SIFs, particularly in the absence of clinical equipoise when intervening in the event of an overdose." There are also feasibility and implementations problems for such RCTs.

We thank the reviewer for this comment and have amended the manuscript to include a statement on RCTs being difficult to implement in the context of SIFs, (page 7).

30. Methods: the dates (start and end) of the study are missing, they are only presented on Figure 1 and should be in the text.

We have amended the manuscript to include study start and end dates. See response to editorial comment.

31. Page 9, line 6: "Previous experience indicates high response rates using these methods." What was the range?

We thank the reviewer for this comment. The cited paper does not actually report response rates, instead simply showing rapid recruitment of a large number of participants. We have amended the manuscript accordingly to read: 'rapid recruitment of a large number of participants' (page 10).

32. Page 12-13, paragraph "Mortality and ambulance-attended non-fatal overdose.": The statistical analysis plan is poorly described. Why did the authors consider at least two

methods (MSM and SCMM)? As they mentioned "e.g." before them, I assume there are other candidates? Would it not be possible to choose one of them know?

Reviewer two highlighted strengths of our statistical plan. We have now amended the statistical analysis section in the manuscript to include a sentence detailing why both MSM and SCMM approaches will be considered in causal inference analyses, but the key differences why one would choose one over the other are a combination of simplicity, flexibility and efficiency (SCMM) vs. a desire to estimate or recover direct causal effects (only MSM) rather than total causal effects (both SCMM and MSM [but MSM tends to be inefficient and complex as outlined]).

"We propose the application of MSM and/or SCMM given key differences between the methods in terms of levels of flexibility, bias and ease of implementation (i.e. handling missing data and dropout, exposure and covariate interactions, estimation for continuous exposures, covariate/confounder history imbalance across exposed and unexposed treatment groups and precision; all favouring SCMM) and the ability to estimate direct longer term (not total) causal effects if required (only possible with MSM)." (page 14)

33. Also, page 12, lines 59-60 "under certain assumptions and conditions": what are these assumptions? Would the data be in verify them?

This part of the sentence has been removed and by "….under certain assumptions and conditions…" we were referring to the range of key differences between the two different causal inference methods. This is now outlined in much greater detail in a subsequent sentence and also directly addresses the reviewer's previous comments regarding why two approaches are to be considered.

34. Page 13: the minimum detectable effect for mortality HR would be 0.3 to 0.42. It seems very high. Have the authors looked for published studies supporting the possibility to reach this level?

Given the nature of the study (prospective cohort) and the clear complexity and challenges associated with participant recruitment and retention (i.e. we are constrained in terms of targeting some form of required notional sample size based on an expected effect magnitude and study design), we appropriately present power/minimum detectable differences given these recruitment and retention constraints, historically observed mortality in this population and expected exposure (MSIR use) distribution. We believe this estimate provides a clear a priori position of the study power to estimate an effect of MSIR use, which can be used to contextualise subsequent study estimates and findings.

35. Similarly, some data could be used to put in perspective the minimum detectable effect on non-fatal OD (e.g. https://pubmed.ncbi.nlm.nih.gov/35690956/)

Thank you for this suggestion. The relationship between self-report data and linked records of non-fatal overdose is unknown and we have reported the minimum detectable effect. We have included reference to the COSINUS cohort in the introductory material.

36. Page 13-14, paragraph "Economic evaluation". Do the authors plan to use modelling to project long-term effect? As they plan to account for long term diseases (e.g. hepatitis C)?

The focus of the economic modelling will be on short term impacts, due to large uncertainty in longer term outcomes associated with a short period of MSIR use. We have modified this section to use person-years lived with hepatitis C as an example, rather than just hepatitis C.

"QALY gains will be estimated based on additional OAT uptake, treatment of comorbidities (e.g. person-years lived with hepatitis C), …" (page 16)

VERSION 2 - REVIEW

**Reviewer**	**2**
**Name**	**Otiashvili, David**
**Affiliation**	**Addiction Research Center, Alternative Georgia**
**Date**	**07-Jan-2025**
**COI**	

I believe all the comments and concerns have been addressed. I did not see the figure in the revision files, but I believe it stays the same as for original submission.

**Reviewer**	**3**
**Name**	**Cousien, Anthony**
**Affiliation**	**Sorbonne-Paris**
**Date**	**20-Jan-2025**
**COI**	

The authors have replied to all my comments. I don't have any remaining concern.
